# Radiation-Induced Attenuation of Perfluorinated Polymer Optical Fibers for Radiation Monitoring

**DOI:** 10.3390/s17091959

**Published:** 2017-08-25

**Authors:** Pavol Stajanca, Katerina Krebber

**Affiliations:** Bundesanstalt für Materialforschung und -prüfung (BAM), Unter den Eichen 87, 12205 Berlin, Germany; katerina.krebber@bam.de

**Keywords:** perfluorinated polymer optical fiber, Cytop, ionizing radiation, radiation-induced attenuation, gamma radiation monitoring

## Abstract

Due to some of their unique properties, optical fiber dosimeters are attractive and extensively researched devices in several radiation-related areas. This work evaluates the performance and potential of commercial perfluorinated polymer optical fibers (PF-POFs) for radiation monitoring applications. Gamma radiation-induced attenuation (RIA) of two commercial PF-POFs is evaluated in the VIS spectral region. Influence of a dose rate and temperature on RIA measurement is investigated, along with defect stability and measurement repeatability. Co-extruded PF-POFs are identified as more suitable for radiation monitoring applications due to lower dose-rate dependence. With co-extruded PF-POF, RIA measurement holds potential for highly-sensitive radiation monitoring with good reproducibility. The results show that operation in the blue part of the spectrum provides most favorable performance in terms of the largest nominal radiation sensitivity, lower temperature, and dose-rate dependence as well as higher defect stability. We demonstrate for the first time to our knowledge, that PF-POFs can be used for distributed detection of radiation with doses down to tens of Grays. The off-the-shelf, user-friendly PF-POF could be of interest as a cheap, disposable sensor for various applications, especially of a more qualitative nature.

## 1. Introduction

Over the last decades, significant attention has been devoted to the development of optical fiber dosimeters [1,2,3]. Gamma radiation is still finding new applications which are no longer limited to the nuclear industry. These include applications in medicine, sterilization, food industry, non-destructive testing, material processing, recycling, and others [4]. Growth of the interest in utilization of gamma radiation also generates a demand for development of new suitable dosimeters. Among available dosimetry technologies, optical fiber dosimeters (OFDs) can offer several advantages, such as a small footprint, electromagnetic immunity, or possibility of remote and real-time measurement. Monitoring of radiation-induced attenuation (RIA) of a fiber is perhaps the most straightforward OFD technique. Radiation damages the material of the fiber which, in turn, degrades the fiber’s transmission properties. The corresponding attenuation increase can then be measured optically and correlated to the total dose that the fiber has been subjected to. The topic of ionizing radiation influence on optical fibers represents a complex and intriguing issue that has been studied extensively for more than four decades [5,6,7]. RIA is associated with the generation of various structural defects in the fiber material and is typically strongly wavelength and composition dependent [7]. Most of the research so far has been focused on the glass optical fibers (GOFs). In particular, phosphorous-doped fibers have been identified as one of the most promising candidates for various RIA-based dosimetry applications [8,9,10,11,12]. Due to the superior temperature stability of their radiation-induced defects, phosphorous-doped GOFs are known for their high radiation sensitivity, linear response, and temperature and dose rate independence at certain wavelengths.

Only more recently, possibility of using polymethyl methacrylate (PMMA) polymer optical fibers for RIA-based radiation monitoring applications has been explored, as well [13,14,15]. Considerable attention has been paid to the development of polymer optical fibers (POFs) in the recent years [16,17]. In comparison to GOFs, large-diameter multimode (MM) POFs are typically more robust, flexible and considerably cheaper. The cost saving factor does not stem only from the lower material cost, but in a large part from the user-friendlier processing and handling of the POFs. Thanks to this, expenses related to high precision equipment and trained personnel typically required for GOF handling can be minimized. POFs can, thus, yield relatively cheap, user-friendly monitoring systems suitable also for industrial environments. In addition, unlike glass fibers, POFs do not fail in a brittle way and have better biocompatibility. Therefore, they are generally more acceptable for medical or even in vivo applications. This is substantiated by a considerable increase of interest in POF-based monitoring system for radiotherapy in the recent years [15,18,19,20].

Our recent study suggested that perfluorinated polymer optical fibers (PF-POFs) might have considerably higher radiation sensitivity than PMMA fibers [21]. PF-POFs are based on a perfluorinated optical polymer Cytop which offers low-loss transmission unmatched by any other polymer material. In addition, low-loss transmission window of PF-POFs covers much broader interval of VIS and NIR wavelengths [22], thus, opening the possibility of RIA monitoring in a wider spectral range. Nevertheless, PF-POF radiation sensitivity increases strongly towards shorter wavelengths [21], and we showed that RIA monitoring of PF-POFs in the VIS region could be promising for radiation sensing [23,24].

In this work, we present results of more detailed and comprehensive investigation of RIA in commercial PF-POFs in order to assess the potential and limitations of their use for RIA-based radiation monitoring. Gamma radiation response of two different types of commercial PF-POF is investigated and compared in the low dose region (<100 Gy). We test reproducibility of the measurement, dependence of fiber’s RIA on a dose rate and temperature as well as potential for distributed radiation measurement. We show that co-extruded type of PF-POFs could be an interesting candidate as a highly-sensitive, cheap, and disposable sensor for various applications, especially of more qualitative nature. In addition, for the first time to our knowledge, we demonstrate the possibility of distributed detection of low-dose irradiation with polymer optical fiber.

## 2. Materials and Methods 

Two different types of PF-POFs are commercially available; fibers drawn from preform produced by Asahi Glass Company (Tokyo, Japan) and co-extruded fibers produced by Chromis Fiberoptics (Warren, NJ, USA). Compared to co-extruded fibers, PF-POFs drawn from preform are known to have a higher degree of purity. Resultantly, they typically exhibit lower attenuation and smoother back-reflection traces [22]. As the impurities and particular manufacturing conditions may influence fiber’s response to gamma radiation, both fiber types are tested and compared. Co-extruded fibers are represented by GigaPOF-50SR [25], while Fontex is used as an alternative representative of PF-POFs drawn from preform [26]. Both fibers have 50 µm graded-index MM core and an outer diameter of around 500 µm. Bare fibers without any additional jacketing were used in this work.

The fiber irradiation was performed at ^60^Co irradiation facility of Helmholtz Zentrum Berlin (HZB). Circularly-arranged set of ^60^Co rods is housed in a lead shielding container and is raised from it for the irradiation procedure. The ^60^Co decays into ^60^Ni by emitting beta particles and gamma radiation with energy of 1.17 MeV and 1.33 MeV. In the presented setup, steal shielding surrounding the ^60^Co rods is used to block the beta particles. The dose rate can be adjusted from roughly 250 Gy/h down to single Gy/h by varying the distance of the sample from the ^60^Co source. The temperature in the irradiation room was stable between 18–21 °C throughout the experiment.

**Figure 1:**
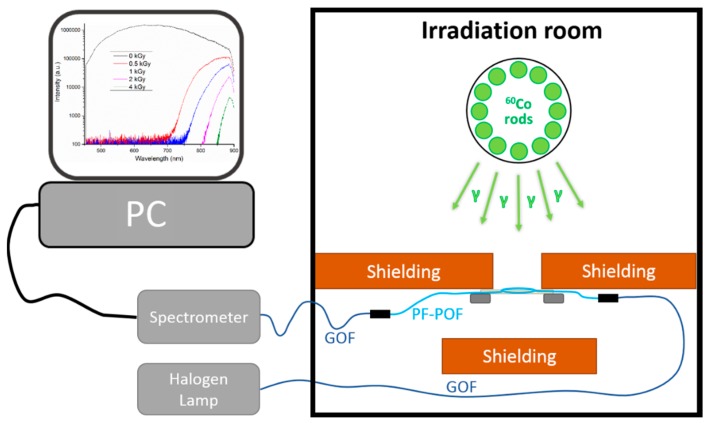
Schematic illustration of experimental setup used for on-line RIA measurement of investigated PF-POFs.

The setup used for the fiber irradiation is depicted in Figure 1. The investigated fibers are cut into roughly 1.5 m long samples. Both ends of the samples were put into F-ST clamp connectors and fiber end faces were polished. The central part of the sample with a length of 1 m was wound into a coil with roughly 7 cm diameter. The coil was fixed onto a thin (2 mm) acrylic plate with the help of small pieces of scotch tape. The plate was held vertically so that the fiber coil is in the position (distance from the ^60^Co source) corresponding to the selected dose rate. The dose rate at given position was verified prior to the fiber irradiation with a calibrated ionizing chamber-based dosimeter. Three different positions 60 mm, 474 mm, and 1774 mm away from the outer wall of the gamma radiation source steal shielding were used. Corresponding dose rates for the three position are 146 ± 2 Gy/h, 15.0 ± 0.1 Gy/h and 1.5 ± 0.1 Gy/h, respectively. For the sake of brevity, simplified dose labels of 150 Gy/h, 15 Gy/h, and 1.5 Gy/h are used further throughout this paper. The coiled fiber samples were placed on the plane plate in the field of the radially emitting radiation source. As a result, different coil sections are at slightly different distances from the radiation source, i.e., are irradiated at different dose rates. The maximal estimate of this dose rate inhomogeneity amounts to ±1.5%, ±0.6%, and 0.15% for irradiation distance of 60 mm, 474 mm, and 1774 mm, respectively.

A fiber-coupled halogen lamp AQ4305 from Yokogawa Electric Corporation (Tokyo, Japan) was used as a source of broadband light, while a computer-controlled CCD spectrometer HR4000 from Ocean Optics (Dunedin, FL, USA) was used to monitor fiber transmission during irradiation. The spectrometer is sensitive in 450–900 nm wavelength range. For each sample, integration time was optimized to yield the maximal initial intensity level that does not saturate the spectrometer. A boxcar smoothing function of the spectrometer was used and set to 10 point smoothing. The transmission spectrum of the sample was saved every 5 s, 1 min, and 5 min for irradiation at 150 Gy/h, 15 Gy/h, and 1.5 Gy/h, respectively. Auxiliary MM glass optical fibers were used to connect the PF-POF sample placed in the irradiation room with the optoelectronics located in the control room. The rest of the PF-POF, connectors, and connecting GOFs were shielded from the radiation by lead bricks. Only 1 m of the PF-POF sample was irradiated during each individual irradiation session.

Prepared samples were irradiated under different conditions and fiber spectral transmission is periodically measured with the spectrometer and logged on the computer (PC). The study is limited to the low-dose region (<100 Gy). The choice of the employed dose rates and the total dose region is given by practical limitations of the used irradiation setup rather than by considerations for a particular application. The fiber RIA (in dB/m) evolution with a total dose D(t) was evaluated from the spectral transmission data as:

(1) $$\mathrm{RIA}\left(\lambda ,D\right)=-\frac{10}{L_{0}}\log\left(\frac{I\left(\lambda ,D\right)}{I\left(\lambda ,0\right)}\right)$$

where I(λ,D) is the recorded transmitted spectral intensity at dose D(t) and $L_{0}$ is the length of the irradiated fiber segment (1 m). The dose values D(t) were calculated based on the used irradiation dose rate and irradiation time.

## 3. Results

### 3.1. Fiber Type Comparison and Dose-Rate Dependence

**Figure 2:**
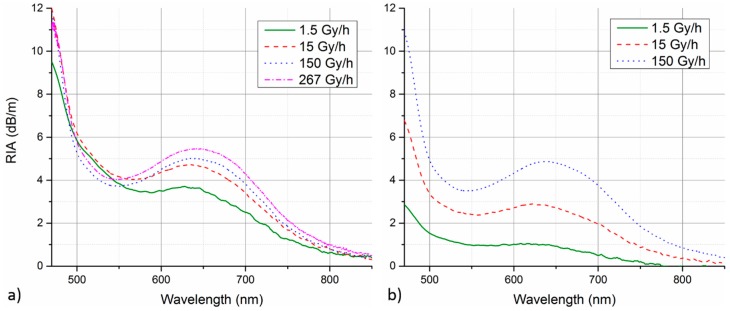
Spectral shape of GigaPOF-50SR (**a**) and Fontex (**b**) RIA measured for irradiation to 100 Gy at different dose rates.

**Figure 3:**
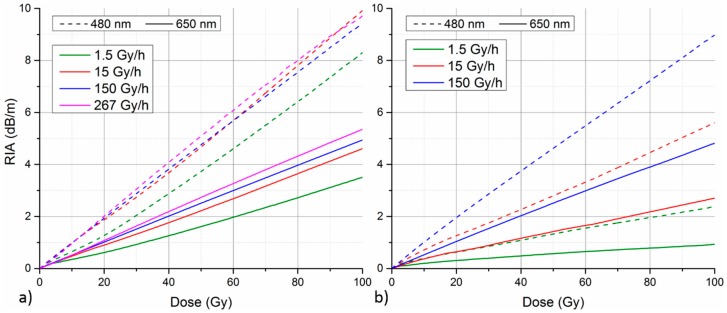
RIA of GigaPOF-50SR (**a**) and Fontex (**b**) PF-POF as a function of total radiation dose measured at different irradiation dose rates. RIA growth at two selected wavelengths is compared.

In the first step, the RIA response of both investigated fibers was measured at different irradiation dose rates. Figure 2 depicts the spectral dependence of the measured RIA for GigaPOF-50SR (a) and Fontex (b) PF-POF obtained for irradiation to 100 Gy at dose rates of 1.5 Gy/h, 15 Gy/h, and 150 Gy/h. In the case of GigaPOF-50SR, additional curve for irradiation at 267 Gy/h from our previous study is added to the graph [24]. The figure shows that RIA in both investigated fibers has similar spectral character. In the VIS spectral region, there is a local sensitivity maximum at around 650 nm from which the induced RIA drops rapidly towards IR wavelengths. There is also a local minimum around 550 nm from which the RIA sensitivity raises steeply towards the UV part of the spectrum. Due to the spectral dependence of RIA, a wide range of radiation sensitivities and dose monitoring ranges can, in principle, be achieved with the PF-POF RIA-based system by suitable selection of the monitoring wavelength. However, for quantitative dosimetry applications, insensitivity of RIA growth to dose rate variations is desirable. Figure 2b reveals that this is not the case for Fontex fiber, where the induced RIA generally decreases by more than factor of three with a dose rate change from 150 Gy/h to 1.5 Gy/h. This could lead to serious errors in system’s dose reading in real-life applications where the dose rate is unknown. The situation is considerably better in the case of GigaPOF-50SR (Figure 2a), especially on the blue side of the monitored wavelength range. This is also visible in Figure 3 comparing the RIA growth at an increasing total dose for the two PF-POFs at different dose rates. The curves for two different wavelengths of highest interest are compared; namely 650 nm as the local RIA sensitivity maximum in the VIS region and 480 nm at the blue edge of the monitored spectra where the RIA sensitivity is strongly increasing. Even though the employed spectrometer is able to measure wavelengths down to 450 nm, its sensitivity drops rapidly towards these shorter wavelengths. In combination with the lower intensity of the employed light source in this spectral region, we were not able to evaluate RIA growth reliably at wavelengths shorter than 480 nm in our experiment. Therefore, 480 nm represents the wavelength of the highest RIA sensitivity in our measurements.

In Figure 3b for Fontex fiber, one can clearly see the strong dose-rate dependence of RIA growth at both selected wavelengths. The RIA sensitivity, i.e., RIA growth rate, is generally higher at 480 nm, as expected from the RIA spectral character (Figure 2b). Fontex RIA response is fairly linear at all investigated dose rates and both selected wavelengths. However, its RIA sensitivity drops strongly with decreasing irradiation dose rate. The strong dose-rate dependence disqualify the fiber from use in most of the radiation monitoring applications. On the other hand, decreased RIA sensitivity of Fontex PF-POF at lower dose rates could be advantageous in applications requiring increased radiation tolerance of the fiber.

The RIA dose-rate dependence can be observed also for the case of GigaPOF-50SR (Figure 3a). However, the magnitude of the effect is considerably smaller. Compared to 650 nm, the influence of dose rate on RIA growth is smaller at 480 nm and also appears to be decreasing at higher dose rates. While the irradiation at 1.5 Gy/h clearly yields considerably slower RIA growth, the curves for the other three dose rates are much more consistent. Considering only these three higher dose rates, operation at 480 nm is clearly superior. The mean RIA sensitivity at 480 nm and 650 nm is 97 ± 2 dBm^−1^/kGy and 51 ± 5 dBm^−1^/kGy, respectively. This mean sensitivity is calculated as an average from partial sensitivities determined from linear regression of the individual RIA growth curves at the considered dose rates (15 Gy/h, 150 Gy/h, 267 Gy/h). The relative deviation of individual RIA growth curves from the mean calibration curve of determined linear sensitivity was evaluated. The relative deviation of the three curves from the mean calibration at 480 nm generally stays within ±6%, while it gets as high as 14% for 650 nm curves.

The presented results clearly show that co-extruded GigaPOF-50SR has much lower RIA dose-rate dependence than Fontex PF-POF. This represents superior performance with regard to gamma radiation monitoring applications. Therefore, we further focus only on GigaPOF-50SR in the following parts of the work.

### 3.2. RIA Annealing and Teperature Dependence

**Figure 4:**
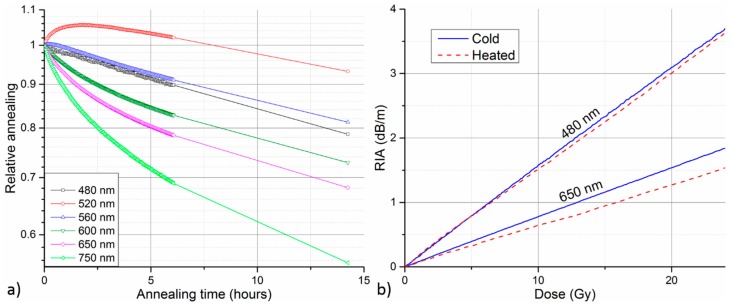
(**a**) Relative annealing of fiber’s RIA at selected wavelengths after irradiation at 150 Gy/h. (**b**) Comparison of fiber’s RIA growth at two selected wavelengths under room (cold, 20 °C) and elevated (heated, 40–50 °C) temperature. Irradiations were performed at a dose rate of 150 Gy/h.

Due to thermally-activated recovery of many of radiation-induced defects in the fiber material, fiber’s RIA response is often temperature dependent and induced RIA anneals over the time after the irradiation is stopped. In order to further asses the fiber’s RIA response and its suitability for radiation monitoring applications, RIA annealing behavior and RIA growth temperature dependence was investigated as well. Figure 4a depicts the fiber’s RIA annealing at selected wavelengths over 14 h after the stop of irradiation at 150 Gy/h. The graph reveals strongly wavelength dependent annealing behavior of the fiber. Wavelengths on the blue edge of the monitored spectrum (<500 nm) exhibit the slowest annealing, indicating higher stability of the corresponding radiation-induced structural defects. For wavelengths between roughly 500 nm and 560 nm, a further increase of RIA is actually observed in the first few hours after the irradiation. Only after that, gradual annealing of RIA sets in. The maximal post-irradiation RIA increase of over 5% was observed after two hours at 520 nm. For wavelengths longer than 570 nm, more rapid RIA annealing of multi-exponential nature is observed. In this spectral region, relative RIA annealing rate increases monotonically with growing wavelength. Comparing two selected wavelengths of high RIA sensitivity (480 nm vs. 650 nm), it is obvious that defects responsible for RIA at 480 nm have higher stability. RIA at this wavelength stays within 95% of its original value for more than 2.5 h after the irradiation end. On the other hand, RIA drops to roughly 85% of its original value at 650 nm in the same time period.

The irradiation facility used in this work do not allow precise temperature control. Nevertheless, temperature influence on GigaPOF-50SR’s RIA response was tested at least in a rudimentary way. This time, fiber coil to be irradiated was fixed onto a front face of an additional lead shielding brick instead of the acrylic plate. The rest of the irradiation setup and geometry remains the same as described in Section 2. Two separate irradiations were performed with such altered sample arrangement. In one case, the brick had a normal temperature of irradiation room (20 °C); second time, the brick was heated with a help of an induction hot plate up to approximately 55 °C before irradiation. It is important to note that POFs are generally not designed to withstand higher temperatures (ca. >80 °C) and, therefore, operation at temperatures much higher than the tested one is not expected. As the heating is not maintained during irradiation, temperature of the brick with the fiber sample gradually drops. Therefore, only 10 min irradiation at 150 Gy/h, corresponding to a total dose of 25 Gy, was performed in this case. Temperature sensor (Almemo FHAD 460, Ahlborn) was placed on the back side of the lead brick to monitor its temperature throughout the experiment. During the irradiation, temperature of the lead brick dropped from initial 51 °C to 40 °C at the end of the irradiation. The efficiency of heat transfer from the lead brick to the fiber sample in our rudimentary setup is not optimized and the precise temperature of the fiber itself is not known. Nevertheless, the performed experiment can illustrate the impact of elevated temperature on fiber’s RIA growth on a qualitative level. Comparison of fiber’s RIA growth at 480 nm and 650 nm from the two measurements at different temperatures is depicted in Figure 4b. A drop of RIA growth rate at elevated temperature could be observed for both wavelengths. However, the magnitude of the effect is considerably lower at 480 nm. While the relative RIA decrease between cold and heated fiber stays below 5% at 480 nm, it reaches up to 20% in case of measurement at 650 nm. 

With regard to OFD applications, both the lower annealing rate and temperature sensitivity of RIA further indicate the superior performance of operation at 480 nm over 650 nm. The reason underlying these observations is the higher stability of the corresponding structural defects in the fiber material. Further insight into fiber’s RIA annealing behavior would require detailed study into the origin and nature of the individual radiation-induced defects, which goes beyond the scope of this work. 

### 3.3. Repeatability

**Figure 5:**
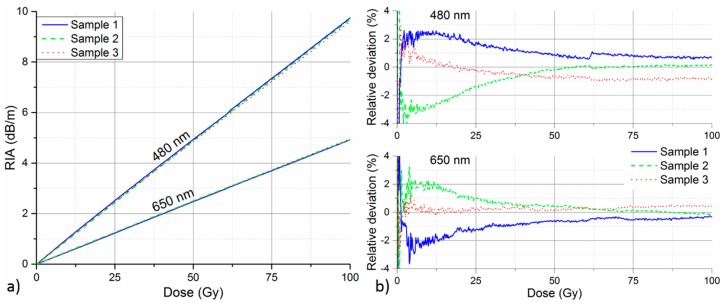
Repeatability of RIA measurement with irradiation of three different samples under the same conditions (room temperature, 150 Gy/h). (**a**) RIA growth curves at two selected wavelengths. (**b**) Relative deviation of the individual RIA curves from the respective mean values at given wavelengths.

As it is crucial for the reliability of the proposed radiation monitoring system, the repeatability of RIA measurement with GigaPOF-50SR was investigated as well. Three fiber samples from different sections of roughly 300 m long spool were prepared and irradiated under the same conditions, i.e., irradiation to 100 Gy at 150 Gy/h under room temperature conditions. Figure 5a depicts the RIA growth at 480 nm and 650 nm for all three samples. The curves at both selected wavelengths are highly consistent, indicating good repeatability of the RIA-based radiation measurement. The individual RIA curves were interpolated on a common dose axis with fine discretization, in order to facilitate calculation of their mean value and relative deviations. Relative deviations of the individual curves from the mean value are presented in Figure 5b, separately for respective wavelengths. The relative deviation generally stays within ± 3% for both wavelengths of interest. A notable exception are very low dose values (<0.5 Gy) where RIA is still relatively small and, therefore, highly sensitive to fluctuations of intensity measurement. As the magnitude of RIA increases, the impact of these fluctuations becomes less significant and relative deviation improves. We would like to note that investigation with samples from different fiber batches would be suitable in order to provide more comprehensive information on measurement reproducibility for practical applications.

### 3.4. Distributed Radiation Detection

There are certain distributed fiber optic techniques, such as optical time domain reflectometry (OTDR) [27], that allow measurement of fiber attenuation profile along the extended lengths of the optical fibers. Monitoring of fiber’s radiation-induced attenuation with such a technique could be employed for distributed radiation measurement [10,28]. Selection of a suitable operation wavelength is crucial for exploitation of the optical fiber system’s potential for the distributed radiation monitoring. The selected wavelength should provide low inherent attenuation, so that monitoring of extended fiber lengths is possible. At the same time, radiation sensitivity at this wavelength needs to be large enough to be suitable for intended application. PF-POFs are known to offer the lowest inherent attenuation among polymer optical fibers. Here we explore the possibility of distributed radiation monitoring with the investigated PF-POF. As an optimal monitoring wavelength, we selected 650 nm. Fiber inherent attenuation is still acceptably low (<100 dB/km) at this wavelength [22]. At the same time, 650 nm corresponds to the local RIA sensitivity maximum of the fiber on the red side of the VIS region (Figure 2a). Higher sensitivity could be achieved at wavelengths close to the UV part of the spectrum. However, operation at these wavelengths would lead to a decrease of possible monitoring length, as fiber attenuation increases towards shorter wavelengths. On the other hand, operation at longer wavelengths would lead to a significant decrease of system sensitivity, because fiber’s RIA sensitivity drops rapidly towards the longer wavelengths.

**Figure 6:**
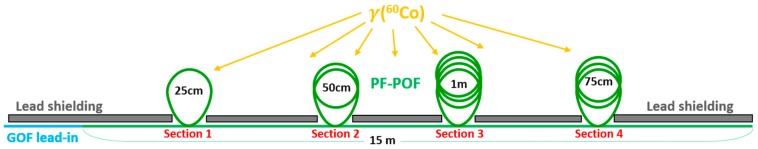
Schematic illustration of experimental configuration used for distributed fiber irradiation and RIA measurement. Four fiber sections to be irradiated are positioned at an equal distance from the ^60^Co source and are irradiated simultaneously under the same conditions.

Figure 6 shows schematic illustration of distributed fiber irradiation. Four separate sections of 15 m long GigaPOF-50SR were irradiated simultaneously to 20 Gy at a dose rate of 150 Gy/h. To test the spatial resolution limit of radiation detection, all sections had different lengths (0.25, 0.5, 1 and 0.75 m). Fiber OTDR trace was measured before and after irradiation with MM OTDR (LOR-200, Luciol Instruments) operating at 650 nm. Pulse duration of 0.25 ns and 5 × averaging was used for the measurements. 

**Figure 7:**
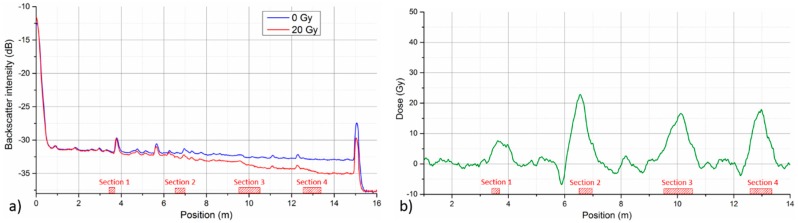
(**a**) Fiber backscattering traces before and after irradiation to 20 Gy. (**b**) Reconstructed dose distribution along the irradiated fiber. Four irradiated sections are indicated by red striped bars on the X-axis.

Figure 7a depicts the fiber’s backscattering traces before and after irradiation. A typical structured nature of backscattering traces of co-extruded PF-POFs is clearly visible. A presence of these minor reflection points is associated with increased level of inhomogeneities and impurities in the fiber core [22]. To recover radiation dose distribution from OTDR measurement, fiber OTDR trace needs to be differentiated and divided by fiber’s RIA sensitivity at given wavelength [10]. Typically, some sort of curve smoothening algorithm is applied before the differentiation. In our case, Savitzky-Golay filtering was used to smoothen the raw OTDR traces [29]. RIA sensitivity of 51 dBm^−1^/kGy determined for 650 nm in Section 3.1 was used in our calculation. In order to avoid artifacts coming from the local scattering centers of co-extruded PF-POFs, described dose reconstruction is performed not only for OTDR trace after irradiation, but for pristine fiber as well. The curve for pristine fiber is then subtracted from the irradiated one, yielding final dose distribution along the fiber depicted in Figure 7b. All four irradiated sections of the fiber can be clearly identified. Moreover, the reconstructed dose values in Sections 2–4 with lengths 0.5 m, 1 m, and 0.75 m, respectively, are within ±20% of the true dose of 20 Gy. The value in the shortest Section 1 (0.25 m) is already considerably underestimated, i.e., roughly 40% of the true dose.

## 4. Discussion and Conclusions

The aim of this work is to perform initial assessment of the potential and limitations of utilization of PF-POFs for RIA-based radiation monitoring. The work is limited to the low-dose region (<100 Gy), dose rates in the 1.5–267 Gy/h range, and wavelengths between 470–850 nm. In the first step, RIA responses of two different commercially-available PF-POF types were compared (Section 3.1). Both fibers exhibited similar spectral character of RIA in the monitored wavelength region and close to linear RIA growth with increasing dose level. While PF-POFs drawn from preform typically provide slightly better optical performance (e.g., lower attenuation, smoother backscattering traces), they appear to suffer from much stronger dose-rate dependence than co-extruded PF-POFs. Obtaining the same dose reading regardless of irradiation conditions is a key requirement for quantitative dosimetry applications. Therefore, the tested PF-POF drawn from preform (Fontex, Asahi Glass Company) that exhibited strong dose-rate dependence could be deemed as rather unsuitable for radiation monitoring applications. In this context, the co-extruded PF-POF (GigaPOF-50SR, Chromis Fiberoptics) exhibited considerably better performance. Dose-rate dependence could still be observed to a smaller extent; however, the results indicate that the magnitude of the effect decreases with the growing dose rate and decreasing wavelength. Moreover, the fiber has rather high radiation sensitivity making it interesting for low dose applications.

We further focused on GigaPOF-50SR’s performance at two wavelengths of high radiation sensitivity, namely 480 nm and 650 nm. With regard to quantitative OFD applications, operation at 480 nm is clearly superior to 650 nm. It offers higher RIA sensitivity (Figure 2a), lower dose-rate dependence (Figure 3a), lower temperature dependence (Figure 4b) and higher post-irradiation stability (Figure 4a). For the three tested dose rates in the 15–267 Gy/h region, a mean sensitivity of 97 ± 2 dBm^−1^/kGy and 51 ± 5 dBm^−1^/kGy was determined for operation at 480 nm and 650 nm, respectively. The maximal relative deviation of the RIA measurements from the ideal calibration curve due to dose-rate dependence was ± 6% at 480 nm and ± 14% at 650 nm. The maximal determined sensitivity of 97 ± 2 dBm^−1^/kGy is more than two orders of magnitude higher than the maximal gamma radiation sensitivity demonstrated for PMMA POF (0.6 dBm^−1^/kGy at 525 nm) [13]. Considerably higher radiation sensitivities, up to thousands of dBm^−1^/kGy, has been demonstrated with phosphorous-doped GOFs in the VIS and UV spectral region [9,11]. However, the fibers used in these studies were experimental custom-made samples with varying dopant concentration. The investigated off-the-shelf PF-POF combines relatively high radiation sensitivity with further advantages of cheaper and user-friendlier robust POFs. One might argue that PF-POFs are considerably more expensive than PMMA POFs and, therefore, do not bring any cost saving. However, having fiber with higher inherent radiation sensitivity means that shorter fiber length is required to achieve the same overall sensitivity of the measurement system. From our experience, more than 100 × larger nominal sensitivity of PF-POFs (compared to PMMA fibers) overcompensates their higher nominal cost.

It is also important to note that 480 nm represents the lower wavelength monitoring limit imposed by our experimental setup. The results indicate that even higher sensitivities could be achieved at lower wavelengths. Already operation at 480 nm allows sub-gray dose resolution. Therefore, PF-POF RIA monitoring at lower wavelengths might have potential even for applications in medical dose region. Along with increasing RIA sensitivity, operation at shorter wavelengths also seems to yield lower dose rate and temperature dependence, which is important for quantitative dosimetry. Further study of fiber’s RIA in the UV and VIS region below 480 nm would be required to gain further insight into fiber’s dosimetry performance.

For the spectral region investigated in our study, we showed that RIA measurement with employed PF-POF could be a viable technique for qualitative radiation monitoring. The technique provides high radiation sensitivity, potential for distributed sensing (Section 3.4) and good repeatability (Section 3.3). However, current repeatability study was limited only to samples from the same fiber batch. Using OTDR operating at 650 nm, we demonstrated the possibility of distributed detection of radiation for dose as small as 20 Gy and length of irradiated fiber section down to 0.25 m (Figure 7). For longer irradiated sections (0.5, 0.75, and 1 m), we have been even able to reconstruct the actual dose value with a fair accuracy. However, practical potential of presented approach for true distributed dosimetry measurement is limited due to more pronounced dose rate and temperature dependence, as well as relatively fast post-irradiation annealing of RIA at 650 nm. Further optimization of the distributed system with respect to operational wavelength is rather problematic. Operation at wavelengths on the blue side of the VIS spectrum could bring higher sensitivity and better dosimetric performance, but at a price of significant shortening of monitoring length, yielding the system rather impractical. On the other side, using longer wavelengths with lower inherent attenuation, one might monitor longer fiber lengths, but fiber’s RIA sensitivity at these wavelengths is considerably diminished [24]. Nevertheless, we showed that the approach can be interesting for on-line distributed radiation leak detection or radiation field profiling along the fiber length. To the best of our knowledge, this was the first demonstration of a distributed low dose detection using polymer optical fiber.

The topic of optical fiber response to ionizing radiation is a complex and complicated issue. Full understanding of RIA growth and annealing requires detailed knowledge of underlying processes on the structural level, which lies beyond the scope of this paper. Fiber’s RIA response could depend on numerous factors, such as irradiation dose rate, total dose level, temperature, energy of the radiation, interrogation wavelength, or intensity of interrogating light. With respect to practical application, characterization of selected monitoring system under all conditions that can be encountered in that particular application would be desirable. In this context, our study still encompasses only a limited set of possible operation conditions and is, therefore, to be viewed rather as an initial evaluation and guideline for PF-POF’s RIA monitoring performance. The results show that monitoring of RIA of co-extruded PF-POFs in the blue end of the VIS spectrum holds the potential for highly-sensitive radiation measurement with good reproducibility. The suitability of the fiber with regard to temperature dependence and post-irradiation stability of RIA needs to be considered with respect to requirements of particular application. While the RIA measurement suffers from notable dose-rate dependence for dose rates below 15 Gy/h, the results indicate that the impact of dose-rate dependence is diminishing for larger dose rates. Finally, for radiation monitoring applications of more qualitative nature, PF-POFs can be used for distributed detection or mapping of radiation with doses down to tens of Grays. Considering that the tested fiber is a relatively affordable, user-friendly, commercial off-the-shelf POF, we believe it could be an interesting candidate in various radiation-related areas.
